# The possible mechanism and research progress of ACE2 involved in cardiovascular injury caused by COVID-19: a review

**DOI:** 10.3389/fcvm.2024.1409723

**Published:** 2024-05-27

**Authors:** Dan Luo, Mengzhe Bai, Wei Zhang, Junnan Wang

**Affiliations:** Department of Cardiology, Second Hospital of Jilin University, Changchun, Jilin, China

**Keywords:** ACE2, cardiovascular diseases, SARS-CoV-2, RAS system, therapy

## Abstract

ACE2 is the earliest receptor discovered to mediate the entry of SARS-CoV-2. In addition to the receptor, it also participates in complex pathological and physiological processes, including regulating the RAS system, apelin, KKS system, and immune system. In addition to affecting the respiratory system, viral infections also interact with cardiovascular diseases. SARS-CoV-2 can directly invade the cardiovascular system through ACE2; Similarly, cardiovascular diseases such as hypertension and coronary heart disease can affect ACE2 levels and exacerbate the disease, and ACE2 dysregulation may also be a potential mechanism for long-term acute sequelae of COVID-19. Since the SARS CoV-2 epidemic, many large population studies have tried to clarify the current focus of debate, that is, whether we should give COVID-19 patients ACEI and ARB drug treatment, but there is still no conclusive conclusion. We also discussed potential disease treatment options for ACE2 at present. Finally, we discussed the researchers’ latest findings on ACE2 and their prospects for future research.

## 1 Introduction

Since early December 2019, the pandemic caused by the SARS-CoV-2 has rapidly spread to various parts of the world, posing an unprecedented public health threat to humanity (1). Various types of COVID-19 vaccines have been developed, including DNA vaccines, mRNA vaccines, and recombinant protein (spike) vaccines (2). However, due to the complexity and diversity of virus variants, it is still difficult to block the invasion of SARS-CoV-2, especially for elderly patients with underlying cardiovascular diseases. Early studies have found that ACE2 is one of the channels through which coronaviruses enter the human body, and ACE2 is also involved in forming a complex network of mechanisms in the body (3). As an important component of the human body, the cardiovascular system cannot ignore the changes that occur after virus infection enters the body. Here we like to make a systematic summary of the discovery, structure, distribution of ACE2 and its participation in various pathophysiological processes of the body. At the same time, we also summarized the probability of various cardiovascular adverse events after COVID-19 enters the body, providing a basis for clinicians to diagnose and treat. We discussed whether ACEI/ARB drugs should be used for patients with underlying cardiovascular diseases. At present, it is widely believed that the use of such drugs will not increase the likelihood of adverse events. Most studies suggest that there is no correlation between the use of ACEI/ARB and the severity/mortality rate of COVID-19 (4–7). Even a retrospective study from China showed that the use of ARB/ACE-I can reduce the risk of septic shock in patients, and the all-cause mortality risk is significantly lower than that of non ARB/ ACEI (5). But a large study published in the American Medical Journal found that using ACE inhibitors or ARBs does not improve clinical prognosis, but may actually worsen it (8). So far, there is no clear conclusion on whether to use RAS system inhibitors. Finally, we have listed the current potential treatment options and the latest developments related to ACE2, providing ideas for future disease and treatment research.

## 2 ACE2- discovery, structure, expression

ACE2 was identified by 5 ’sequencing of a human heart failure vessel cDNA library by M Donoghue et al. (9) It is the first known homolog of human angiotensin-converting enzyme (ACE), and the relative conservation of exons/introns between ACE2 and ACE catalytic domains further indicates that these two genes were replicated by a common ancestor. ACE2 has distinct signal peptides, single metalloproteinase active sites, and transmembrane domains (10). ACE2, as a type I transmembrane protein, consists of 805 amino acids and has an extracellular N-terminus domain and an intracellular short C-terminus tail. The N-terminus domain has an active site, the zinc metalloproteinase domain (HEMGH domain), which is crucial for the binding of vasodilator peptide Ang 1-7 and SARS-CoV-2 S protein (9). ACE2 is very similar to ACE, especially in the catalytic region. Through comparison, 42% of the metalloproteinase catalytic domains of ACE2 and ACE were the same, which can be used to hydrolyze circulating vascular activity and other regulatory peptides (9, 10). But unlike the ACE gene located on human chromosome 17, the 40 kb ACE2 gene is located on chromosome Xp22 and contains 18 exons. ACE2 only has one catalytic domain, while somatic ACE contains two active sites (11). ACE and ACE2 are both zinc metalloproteinases, and their main functional catalytic difference lies in their substrate specificity, thus their respective physiological roles are different. Essentially, ACE2 is a zinc monocarboxypeptidase that removes a single C-terminal amino acid from sensitive peptides, while ACE removes C-terminal dipeptides (peptidyldipeptidase activity) from its various substrates. Therefore, these two enzymes play distinct roles in the metabolism of their main metabolic pathway RAS and other regulatory peptides (10). Meanwhile, ACE2 does not cleave bradykinin, which further distinguishes ACE2 from ACE (11, 12).

The expression of ACE2 gene was initially discovered in the heart, kidneys, and testes, but subsequent studies have shown that it's distribution is much wider, including the upper respiratory tract, lungs, intestines, and liver (11). Li et al. (13) found through big data processing that ACE2 expression levels were highest in the small intestine, testes, kidneys, heart, thyroid, and adipose tissue; the lowest in blood, spleen, bone marrow, brain, blood vessels, and muscles; It exhibits moderate expression levels in the lungs, colon, liver, bladder, and adrenal glands. At the same time, by evaluating the immune signature enrichment levels in tissues, it was found that ACE2 was not differentially expressed between males and females or between young and elderly people in any tissue. But in the skin, digestive system, brain, and blood vessels, the expression level of ACE2 is positively correlated with the immune characteristics of men and women.

The latest research has identified a novel isoform expressing ACE2 in the airway epithelium (the main site of SARS-CoV-2 infection), which is a truncated variant that is upregulated after interferon treatment. This so-called δ subtype is expected to reduce susceptibility to viral infection due to the lack of SARS-CoV-2 spike high affinity binding sites (14). This variation is mainly limited to the lung airway epithelium and the liver bile duct epithelium. Whether the physiological role and expression of this subtype are directly related to SARS-CoV-2 infection or disease prevention remains to be elucidated.

## 3 The possible mechanism of ACE2 involvement in cardiovascular system injury induced by SARS-CoV-2 virus

### 3.1 ACE2 as a channel for SARS-CoV-2 virus to enter the body

The four main structural proteins of SARS-CoV-2 virus are spike surface glycoprotein (S), small envelope protein (E), matrix protein (M), and nucleocapsid protein (N). The spike surface glycoprotein S initiates the viral infection process by binding to the host cell functional receptor-ACE2 (15). The spherical receptor binding subunit S1 domain of the S protein attaches the virus to the cell receptor ACE2 on the host cell. Subsequently, the rod-shaped fusion subunit S2 domain is cleaved by the host transmembrane serine protease TMPRSS2, and the anchored S2 domain is activated to trigger the double layer fusion of the virus and host lipid, releasing the viral ribonucleoprotein complex into the cell (16) (Supplementary Figure S1).

ACE2 receptors are distributed throughout various organs of the body, especially on the cell surfaces of organs such as the lungs, heart, vascular endothelium, and intestines, where they are highly expressed (11). SARS-CoV-2 targets ACE2 in cells or tissues, which increases the risk of virus entry if overexpressed in the respiratory tract; high expression in the heart and blood vessels is a possible mechanism by which the virus directly damages the heart and blood vessels (17). ACE2 is mainly expressed on the cell membrane and has almost no localization in the cytoplasm. Under normal circumstances, ACE2 is not easily internalized. However, experiments have shown that after SARS-CoV2 infection, receptor ACE2 transfers from the plasma membrane to the cytoplasmic region after treatment with spike protein (17, 18). Therefore, in addition to its membrane binding form, ACE2's soluble form (sACE2)can be detected in plasma and urine through its extracellular domain detachment (19).

Wang et al. (20) evaluated the changes in sACE2 in virus infected individuals and found that soluble ACE2 increased in COVID-19, but was not associated with disease severity or mortality. In contrast, the increase in sACE2 during repeated sampling is independently associated with an increased risk of death, as well as the incidence of acute myocardial injury and circulatory shock.

### 3.2 ACE2 participates in regulating the balance of the RAS system

In classic RAS, renin from the kidney acts on hepatic angiotensinogen to produce angiotensin I (Ang I). Subsequently, ACE catalyzes Ang I to angiotensin II (Ang II). Ang II works by binding to the type 1 receptor (AT1R) and type 2 receptor (AT2R). It is called ACE/Ang II/AT1R axis (21). This axis can mediate a series of biological reactions such as vasoconstriction and elevated blood pressure.

ACE2 is also the main component that maintains the homeostasis of the RAS system (Supplementary Figure S1). Although ACE2 shares considerable homology with ACE, their physiological roles are different, because ACE2 is considered a negative regulator of RAS (22). ACE2 can cleave Ang II to produce Ang 1-7, thereby reducing Ang II levels. At the same time, it can cleave Ang I to produce Ang 1-9, which is further catalyzed by ACE to form Ang 1-7. Finally, Ang 1-7 can activate the MAS receptor (MASR) that promotes vasodilation and anti-inflammatory effects, antagonizing the ACE/Ang II/AT1R axis (9, 23). Ang 1-7 activated MASR triggers downstream signaling pathways, including arachidonic acid release and activation of phospholipase A3, NOS, PI4 K/Akt, MAP kinase, RhoA, and cAMP/PKA. MASR also acts as an antagonist of AT1R, and Ang 1-7/MASR conduction inhibits Ang II effects, including Ang II induced excessive production of reactive oxygen species (ROS), cell apoptosis, MAPK and c-Src phosphorylation, ultimately leading to TGF-β 1,collagen production, ICAM-1, VCAM-1, and MCP-4 expression (22).

Once ACE2 is occupied by the virus, ACE2 lacks degradation, and free Ang II accumulates in the plasma. Excessive Ang II binds to its receptor AT1R, activating the RAS system and generating a series of inflammatory reactions (11). Meanwhile, Ang II increases TNF-α Invertase (TACE) (i.e., ADAM17) activity to inhibit ACE2. In people with heart failure (HF), an increase in plasma ACE2 activity may indicate a loss of ACE2's protective effect in the heart (18, 24). In summary, the accumulation of Ang II, inflammatory cytokines, and SARS coronavirus infection upregulates ADAM17 activity, forming a harmful positive feedback loop, leading to impaired ACE2 mediated organ protection (18, 24).

Osman et al. (25) analyzed the peripheral blood of COVID-19 patients. They found that the expression of ACE2 mRNA decreased compared to healthy patients in circulating blood cells. This may be the result of Ang II's carefully planned feedback regulation loop. Ang II induces ACE2 shedding by promoting AMAD17 activity as a positive feedback mechanism, thereby promoting the loss of its negative regulatory factor ACE2 (24). According to previous reports, overexpression of Ang II reduced ACE2 mRNA production in rat cardiomyocytes and fibroblasts. As the disease progresses, it has been found that inhibiting ADAM17 and enhancing Ang 1-7/MASR axis have potential effects in combating SARS-CoV2 infection (20).

### 3.3 ACE2 participates in regulating apelin

Apelin is a peptide encoded by the apelin gene, located on the long arm of the X chromosome. It is synthesized as a pre pro apelin composed of 77 amino acids, which is cleaved by endopeptidase into biologically active small C-terminal fragments of 13–36 amino acids in length (Apelin-13, Apelin-16, Apelin-17, Apelin-19, and Apelin-36). Apelin and its receptor APJ are widely expressed throughout the cardiovascular system (26, 27).

Lee et al. (28) provided the first evidence of the cardiovascular effects of apelin. They reported a significant instantaneous decrease in systolic and diastolic blood pressure after intravenous infusion of apelin in anesthetized male Wistar rats. In a similar experimental setup, another group observed three different apelin peptides (apelin-12, apelin-13, and apelin-36), which also had antihypertensive effects. Their efficacy was inversely proportional to the molecular weight of the peptide, and the possible mechanism was to trigger the release of vasodilators (such as nitric oxide) from endothelial cells to cause endothelial dependent vasodilation (29).

Meanwhile, apelin is also an endogenous regulatory factor of RAS, which negatively regulates the RAS system by binding to its receptor APJ and antagonizing Ang II activity (26) (Supplementary Figure S2). ACE2 is responsible for some apelin metabolism. Research has shown that ACE2 has the ability to cleave and inactivate [Pyr]—appelin-13 and apelin 17.Between them, [Pyr]—appelin-13 is more susceptible to ACE2 degradation and metabolition than apelin-17 (30).

In addition, apelin is also a positive regulatory factor for ACE2, which can stimulate ACE2 transcription and its enzyme activity (31). The levels of ACE2 mRNA and ACE2 protein in the myocardium of apelin KO mice decreased, which can be saved by infusion of Apelin-13. Meanwhile, Sato (31), Siddiquee (32), Zhang (33) and others found that apelin signaling through apelin receptors specifically increases the activity of the ACE2 promoter, leading to an increase in ACE2 mRNA and protein. These effects are consistent with the ability of [Pyr]—appelin-13 peptide to negatively regulate superoxide production, myocardial hypertrophy, dysfunction, and fibrosis Ang II mediated (33). The downregulation of ACE2 in COVID-19 may weaken the effect of apelin, and the reduced apelin in turn negatively regulates ACE2, leading to the accumulation of Ang II in the body, thereby exacerbating the damage caused by the virus to the body.

### 3.4 ACE2 participates in KKS system

ACE and ACE2 are also closely related to KKS. KKS regulates the opposite effect on RAS by inducing arterial vasodilation (34) (Supplementary Figure S3). KKS is composed of kininogen, kallikrein, prokallikrein, kininase, and bradykinin. High molecular weight kininogen (HK) in plasma releases the active substance kinin under the action of kallikrein. Bradykinin (BK) is the main kinin substance under physiological conditions, and it is also a type of peptide that has been extensively studied. Subsequently, the pre kallikrein further cleaves BK into the active metabolite des Arg9-BK (DABK), and the remaining lysozyme kininogen (cHK) is stable in plasma and can be used as a biomarker for KKS activation (35).

Kinin exerts its biological activity by activating kinin receptors. The vasodilation effect of BK is mainly mediated by B2R, which is abundant in the vascular endothelium and constitutively expressed in most tissues. The activation of B2R can cause the cascade of nitric oxide synthase (NOS), leading to the synthesis of nitric oxide (NO) and cGMP. BK and its active metabolite DABK can also stimulate B1R, which is rarely expressed in healthy tissues but damaged and inflammatory tissue, playing a role in chronic pain and inflammation (36, 37). The activation of B1R and B2R mediates a large amount of vascular permeability and inflammation, leading to a significant increase in the levels of inflammatory cytokines such as IL-1, IL-2, IL-6, IL-8, and TNF-α.They are related to the cytokine storms observed in SARS-CoV-2 ARDS (38–40). At the same time, KKS is also involved in regulating tissue repair, cell proliferation, and antiplatelet aggregation (39).

ACE is the main endovascular peptidase of BK, producing DABK and several inactive intermediates, including BK1–5. ACE2 inactivates DABK by cleaving its C-terminal residue, but has no effect on BK (41). BK receptors are also isomerized with angiotensin receptors AT1R, AT2R, and MASR, forming stable heterodimers such as AT2R-B2R heterodimers, leading to G α (q) And G α (i).The activation of proteins increases, strengthens the production of NO and cGMP, and antagonizes the RAS system (42–44). Meanwhile, researchers have found that ACEI can also be attributed to the intracellular signaling cascade that prevents B2R desensitization through inhibiting the degradation of BK and DABK (40).

### 3.5 ACE2 participates in the immune system

Previous studies have found that ACE2 also has immune regulatory properties of reducing Ang II, through its directly and indirectly interaction with macrophages, which is beneficial for inflammation (45). Recently, another mechanism of SARS-CoV-2 mediated cytotoxic lymphocyte(CTL) inhibition has been discovered, which is driven by the binding of spike proteins to ACE2. Cassioli et al. (46) found that ACE2 is expressed on human CD8 *T* cells during their differentiation into CTLs. ACEI inhibits the assembly of functional immune synapses (IS) when binding to spike proteins in experimental environments. In CTLs, IS serves as the focal point for its cytotoxic effector exocytosis, which is encapsulated in specialized lysosomes called lytic granules rich in perforin (porogen) and granzymes (proteolytic enzymes). Although the spike molecular determinants recognized by these antibodies may potentially bind to other surface receptors, these findings still indicate that the inhibitory effect of the spike on CTL is mediated by ACE2. Onnis et al. (47) found that human quiescent CTLs that do not express ACE2 are not affected by spikes, but after ACE2 forced expression, spike induced IS defects were observed in CTLs. This result supports the above viewpoint, which may be due to the activation of integrins. In addition, surface ACE2 also enhances cell adhesion and regulates integrin signaling (48).

## 4 The interaction between SARS-CoV-2 infection and cardiovascular damage

COVID-19 patients may exhibit different manifestations of cardiovascular injury. Some patients consider cardiovascular symptoms such as palpitations and chest distress as their initial symptoms (49). Meanwhile, many clinical data indicate that susceptibility to SARS-CoV-2 infection and the results of COVID-19 are closely related to preexisting cardiovascular disease (CVD) (Supplementary Table S1). In the confirmed cases of SARS-CoV-2 infection reported by the National Health Commission (NHC) in China, some patients first seek medical attention due to cardiovascular symptoms, rather than respiratory symptoms such as fever and cough. Among the population reported by NHC to have died from COVID-19, 11.8% of patients without underlying cardiovascular disease experienced severe heart damage due to SARS-CoV-2 infection, elevated cTnI levels during hospitalization, and even cardiac arrest (49).

### 4.1 Myocardial injury

Evidence of acute myocardial injury is common among COVID-19 hospitalized patients. In a summary analysis of 11,685 hospitalized patients, 278 cases experienced acute myocardial injury in the hospital and were associated with a higher risk of death (50). Among the 41 patients infected with SARS-CoV-2 at the beginning of the epidemic (51),There were 5 cases of acute myocardial injury associated with SARS-CoV-2, with a significant increase in hypersensitive troponin I (hs cTnI). Subsequently, a study analyzed the clinical characteristics of 138 SARS-CoV-2 infected patients in Wuhan City (52), among all patients, hypertension [43 (31.2%)] and cardiovascular disease [20 (14.5%)] are the most common clinical comorbidities. Inciardi et al. (53) found that a patient without a history of cardiovascular disease developed severe left ventricular dysfunction and acute myocarditis after SARS-CoV-2 infection. The patient's electrocardiogram showed diffuse ST segment elevation; Elevated levels of high sensitivity troponin T and NT-proBNP were detected in the serum; Cardiac magnetic resonance imaging shows increased wall thickness with diffuse biventricular dysfunction, especially in the apical segment, as well as severe left ventricular dysfunction (left ventricular ejection fraction of 35%).

In addition, evidence of direct virus invasion of myocardial cells and cardiovascular endothelium was found in autopsy cases of COVID-19 patients (54, 55), the autopsy results showed that 62% of all deaths occurred with SARS-CoV-2 heart infection (20, 56). Sala et al. (57) reported the first direct evidence of myocarditis through endomyocardial biopsy (EMB) of COVID-19 patients. EMB shows diffuse inflammatory infiltration of T lymphocytes with significant interstitial edema and localized focal necrosis.

In animal experiments, it was also found that mice infected with SARS-CoV-2 exhibited ventricular dilation, myocardial fiber disruption, inflammatory infiltration, fibrosis of the epicardium and interstitium (6). Viveiros et al. (58) also found in animal experiments that the nucleocapsid of the virus can be detected in the hearts of SARS-CoV-2 infected animals, and areas of increased mononuclear infiltration and focal fibrosis appeared in the myocardium of infected hamsters, which were not found in the control group animals. The cardiotoxicity caused by SARS-CoV-2 infection has been confirmed in 3D cardiac tissue models and animal experiments. SARS-CoV-2 infection and its induced cytotoxicity and apoptosis eliminate myocardial cell beating. RNA sequencing confirmed upregulation of genes related to viral infection, interferon signaling, cell apoptosis, and reactive oxygen species stress pathways (6, 59). Importantly, after infection with SARS-CoV-2, viral spike proteins and viral particles were detected in live human heart slices. Further observation of coronavirus particles in myocardial cells of COVID-19 patients (59).Arun Sharma et al. (60)demonstrated that human induced pluripotent stem cell derived cardiomyocytes (hiPSC-CMs) can transfect SARS-CoV-2 *in vitro*. Immunostaining with SARS-CoV-2's unique dsRNA intermediate and “spike” capsid protein confirmed that the expression of ACE2 on the surface of cardiomyocytes allows SARS-CoV-2 to enter, replicate, and produce multiple copies of the virus to infect other cells. Vitro experiments have shown that hiPSC-CM undergo functional changes while being infected by the virus, including decreased contractility and cell arrest. Therefore, at least in theory, there is a potential for the virus to directly affect the heart. Researchers speculate that the mechanism of acute myocardial injury caused by SARS-CoV-2 infection may be related to ACE2. ACE2 is not only widely expressed in the lungs, but also in the cardiovascular system. Therefore, ACE2 related signaling pathways may also play a role in heart injury.

### 4.2 Hypertension

The first case series study in China shows that hypertension is the most common disease among COVID-19 patients, accounting for 27%–30%, while the representativeness of other comorbidities is much lower (61). An observational study included 12,594 patients in New York City, and the results showed that the incidence of hypertension was 34.6% (4). But in fact, the association between COVID-19 and hypertension does not seem surprising, nor does it necessarily imply a causal relationship, as the global incidence of hypertension is high. Interestingly, a retrospective single center cohort study (62) at the Seventh Hospital in Wuhan, China showed that the parameters related to death and respiratory distress are not hypertension, but rather an increase in systolic blood pressure (SBP) values. High SBP was identified as a covariate in mortality and survival prediction models (62). Elevated SBP may be a biomarker of subclinical organ damage (HMOD) mediated by hypertension, making it an important comorbidity factor. Higher SBP may also be due to insufficient or uncontrolled hypertension treatment, or it may be the result of a decrease in ACE2 activity caused by higher SARS-CoV-2 load binding.

Some experimental and clinical studies have shown that ACE2 deficiency may be a pathogenic factor for hypertension (11). ACE2 deficiency is associated with hypertension caused by Ang II. During the development of salt sensitive hypertensive mice, selective knockdown of ACE2 was found to slow down the increase in blood pressure and maintain ACE2 activity (63). These research findings support the important role of ACE2 in maintaining healthy blood pressure. ACE2 lacks overactivation of the RAS system, which may trigger cellular and vascular contractions by promoting inflammatory responses and cytokine storms, as well as stimulating the NADH/NADPH oxidase system (64). In addition, studies have found that increased expression of ACE2 can prevent hypertension, and overexpression of ACE2 by lentivirus leads to increased expression of RAS antihypertensive components and alleviates elevated blood pressure. Recombinant human ACE2 (rhACE2) pretreatment can prevent hypertension caused by Ang II, reduce plasma Ang II, and increase plasma Ang 1-7 levels (11).

### 4.3 Acute coronary syndrome

Crucial to the pathogenesis of acute coronary syndrome is the formation of thrombi at the affected plaque site. The contributing mechanism of thrombosis includes systemic proinflammatory cytokine response, which is the mediator of atherosclerosis and directly leads to plaque rupture through local inflammation, procoagulant factor induction and hemodynamic changes (65). In fact, inflammatory markers can predict the outcomes of acute vascular events. Studies have found that hereditary ACE2 deficiency is related to the up regulation of atherogenic putative mediators, and enhances the responsiveness of proinflammatory stimuli. It suggests that ACE2 plays a key role in inhibiting vascular inflammation and atherosclerosis (11). Viral infection not only causes endothelial dysfunction, but also activates the RAS system by downregulating ACE2, inducing coronary artery vasoconstriction, leading to impaired myocardial perfusion (66).Severe infections often lead to increased metabolic demand and hypoxia in the myocardium, which may increase the already damaged myocardial ischemia and hypoxia situation (65). It is worth noting that MI increases ACE2 mRNA expression in human, mouse and rat hearts.genetic ACE2 deficiency leads to myocardial infarction induced cardiac dysfunction, increasing infarct size and neutrophil infiltration in the surrounding area, upregulation of interferon γ, IL-6 and chemokine MCP-1 (monocyte chemoattractant protein-1), activation of MMP-2/MMP9 and deterioration of extracellular matrix destruction (67, 68).On the contrary, overexpression of ACE2 and the effect of Ang 1-7 improved MI induced cardiac remodeling (11).

### 4.4 Arrhythmias

Arrhythmias are also believed to be related to SARS-CoV-2. In the early stages of the epidemic outbreak, researchers found that 44 out of 170 heart injury patients had arrhythmias in a retrospective cohort study of 1,284 severe COVID-19 patients in Wuhan (69). Research shows that the total incidence of arrhythmia in COVID-19 patients in China is 7.16% (52, 70). The currently recognized view is that the arrhythmia in COVID-19 patients may be caused by electrolyte and hemodynamic disturbances caused by inflammatory stress, and the electrolyte imbalance caused by the interaction between SARS-CoV-2 and the RAS system. The imbalance between them can lead to hypokalemia, thereby increasing the risk of arrhythmia (52).

Among the 138 patients infected with SARS-CoV-2 at the beginning of the epidemic, 16 patients developed new arrhythmias [16 (44.4%)] (52).Anjali Bhatla,BA et al (71) analyzed and studied 700 patients admitted to COVID-19. The study identified 53 arrhythmia related events, including 9 cases of cardiac arrest, 25 cases of atrial fibrillation, 9 cases of bradycardia and chronic arrhythmia, and 10 cases of non persistent ventricular tachycardia. Except for cardiac arrest, all three types of arrhythmia are independently associated with acute mortality. The research results indicate that the incidence of cardiac arrest in COVID-19 patients is related to the severity of the disease, but it is not the only consequence of viral infection.

### 4.5 Heart failure

Clinical studies have found that HF is the most common cause of death among 113 patients who died from COVID-19 after acute respiratory distress syndrome and sepsis (70, 72). Alexander et al. (73) constructed a rabbit model infected with coronavirus. They found that rabbits developed dilated cardiomyopathy after being infected with the virus, exhibiting increased cardiac weight, biventricular dilation, cardiomyocyte hypertrophy, myocardial fibrosis, and histopathological signs of interstitial fibrosis. Similarly, in the mouse experimental model, ACE2 gene knockout mice experienced left ventricular systolic dysfunction and heart failure, with a more significant decrease in ejection fraction (56). The ACE2 gene knockout mice developed left ventricular systolic dysfunction and heart failure, with a more significant decrease in ejection fraction. Overexpression of ACE2 gene improves left ventricular diastolic function in experimental models by reducing reactive oxidative stress, fibrosis, and myocardial hypertrophy (74, 75).

The lack of ACE2 increases the susceptibility to heart failure. Ang II participates in regulating cardiac hypertrophy by stimulating three main MAP kinase pathways (ERK1/ERK2, p38, and c-Jun NH2-terminal kinase or JNK). ERK1 and ERK2 are stimulated by Ang II in myocardial cells and play a major role in cardiac hypertrophy by regulating the expression of ANP (76).The enlarged heart releases natriuretic peptides ANP and BNP, ANP directly inhibits muscle cell growth and indirectly reducing hemodynamic load through its diuretic and natriuretic properties. At the same time, ANP released by hypertrophic hearts may block Ang II/ET-1 to mediate ACE2 mRNA reduction by increasing cGMP, reducing ERK1/ERK2 activity, and increasing MKP1 (77).

Recently, a study was published measuring circulating levels of ACE2 in a European population of 1,485 males and 537 females with HF. Among them, 80 HF patients had elevated plasma ACE2 levels (78). Interestingly, the strongest predictor among the two cohorts was male, which is consistent with the increased prevalence and severity of COVID-19 in males (75).

### 4.6 Coagulation defects

The COVID-19 pandemic may affect the prevention and management of thrombotic and thromboembolic diseases in various ways. It has been reported that the incidence of venous and arterial thrombotic complications in critically ill patients with COVID-19 admitted to ICUs in three hospitals in the Netherlands is very high. It is found that the cumulative incidence rate is 31%, of which pulmonary embolism (PE) is the most commonly diagnosed thrombotic complication. It was also observed that anticoagulation therapy at baseline prevented thrombotic complications, but not all cause of death, which may support the hypothesis of *in situ* immune thrombosis (79).In a cohort study in China, the medical records of 191 adult COVID-19 patients showed that 27 out of 54 non surviving cases (50%) developed coagulopathy, while 10 out of 137 surviving cases (7%) developed coagulopathy (80).Tang et al. (81) described the results of 183 patients admitted to Wuhan Tongji Hospital. 71.4% of non survivors and 0.6% of survivors showed clear evidence of disseminated intravascular coagulation (DIC), with a median time of 4 days for DIC testing.

In the experimental model of thrombosis, the expression of ACE2 was detected in the thrombus extract, increasing the possibility that ACE2 may play a role in regulating circulating platelet thrombosis and hemostasis function. Research has found that activating the ACE2/Ang 1–7/MASR pathway with ACE2 activator (XNT) can reduce Ang II and demonstrate antithrombotic activity in animal models (82). It indicates the crucial role of ACE2 in the coagulation pathway.

ACE2 may exert anti-thrombotic effects through various mechanisms, the most important of which is the renin angiotensin pathway. The decrease in ACE2 leads to an increase in Ang II, which stimulates the expression of plasminogen activator inhibitor 1 (PAI-1) in a dose-dependent manner in various cells, including smooth muscle cells, endothelial cells, and adipocytes. PAI-1 is the main inhibitor of the plasma fibrinolysis cascade reaction, therefore, the activation of the renin angiotensin system can significantly promote the pre thrombotic state (83). The decrease in ACE2 activity can also increase vascular permeability, leading to the expression of tissue factor (TF) in subendothelial cells, as well as white blood cells and platelets, triggering coagulation, thrombosis, and disseminated intravascular coagulation. Cross talk between KKS and the coagulation system has been discovered, and the activation of factor XII by kallikrein may contribute to the procoagulant state (84).

### 4.7 Hypertension and coronary heart disease affect ACE2 levels

According to reports, a decrease in cardiac ACE2 levels in hypertension and cardiovascular disease has been found to be associated with high-risk severe COVID-19 (85). The reason may be due to excessive activation of the pro-inflammatory ACE/Ang II/AT1R axis, triggering the cancellation of overexpression of the ACE2 pathway. People speculate that ACE2 deficiency may play a central role in the pathogenesis of SARS-CoV-2 infection. Virus invasion induced down-regulation of ACE2 may be particularly harmful to individuals with baseline ACE2 deficiency, for example, due to older age, diabetes, hypertension and previous heart disease, including heart failure (86).

Mild or moderate angiotensin converting enzyme deficiency seems unlikely to prevent virus invasion, as SARS-CoV-2 has an inherent high affinity for ACE2 receptors (16). In contrast, virus induced downregulation of ACE2 may amplify the ACE/Ang II/AT2R axis (unfavorable) and ACE2/Ang 1-7/MASR axis (protective). At the lung level, dysregulation will greatly promote the progression of inflammation and hypercoagulant processes, which depend on the high activity of local Ang II and the relative low activity of Ang 1-7. This series of events may also be accompanied by other mechanisms, including impaired immune response to initial viral invasion, or genetic susceptibility to excessive inflammation and thrombosis (86).

Tucker et al. (87) conducted extensive mononuclear RNA Seq sequencing on the left ventricle of 2 patients with dilated cardiomyopathy, 11 patients with hypertrophic cardiomyopathy, and 15 patients without heart failure. The results showed that the expression of ACE2 in myocardial cells was upregulated in dilated cardiomyopathy and hypertrophic cardiomyopathy, thus confirming that previous cardiovascular diseases were the main driving factors for the increase in myocardial cell specific ACE2 transcription.

### 4.8 ACE2 in long-term COVID-19 cardiovascular system

SARS-CoV-2 infection not only causes short-term damage to the body, but also long-term chronic damage. Because it has been found that patients who have recovered from SARS-CoV-2 infection have an increasing risk of death and the development of new lung, nervous system, metabolic, gastrointestinal, and cardiovascular diseases within 6 months (88).The World Health Organization (WHO) defines this long-term injury that occurs after acute infection as long-term COVID, which refers to individuals who have been affected by daily function for at least 3 months since SARS-CoV-2 infection, with symptoms lasting for at least 2 months, and acute sequelae that cannot be explained by other diagnoses (89). The long-term cardiovascular symptoms of COVID include hypertension, arrhythmia, chest pain, coronary atherosclerosis and heart failure (90). A study has reported the results of myocardial MRI evaluation in COVID-19 rehabilitation patients, which showed the presence of myocardial edema, necrosis, and fibrosis (91) and about 40% of these abnormalities are not related to myocardial ischemia (92). Recurrent arrhythmias are believed to be associated with decreased cardiac reserve, corticosteroid use, and dysregulation of RAAS. Dysregulation of cytokines (IL-6, IL-1, and TNF-α)can prolong ventricular action potential by regulating the expression of ion channels in myocardial cells, ultimately leading to permanent arrhythmia (93).Some researchers have suggested that sustained dysregulation of ACE2 in tissues may be a potential mechanism for long-term COVID (93). Although there is no clear evidence to prove it, it cannot be denied that the imbalance of ACE2 levels and dysfunction leading to elevated levels of inflammatory cytokines in the body are one of the reasons for long-term damage to the cardiovascular system. The critical protective function of ACE2 in the cardiovascular system should not be underestimated, as the first phenotype discovered in Ace2 mutant animals is impaired cardiac function (1).

## 5 Whether to use ACEI and ARB drugs

Hypertension patients infected with SARS CoV-2 had a debate when receiving antihypertensive drugs: whether we should give them ACEI and ARB drugs when treating COVID-19, and whether the use of ACE blockers will increase the risk of COVID-19 infection and exacerbate disease progression. There is currently no clear evidence to support the use of ACEI/ARB drugs in cardiovascular patients diagnosed with COVID-19. In a large study published in the American Medical Journal, in this trial, starting the use of ACE inhibitors or ARBs in adult critically ill patients with COVID-19 did not improve clinical prognosis and may actually worsen clinical outcomes (8).But another part of the research suggests that there is no association between the use of ARB/ACE-I and the severity/mortality rate of COVID-19 (24.7% vs. 24.8%, 95% CI −3.5 to 3.5) (4). A retrospective study from China showed that the use of ARB/ACE-I can reduce the risk of septic shock in patients, and the all-cause mortality risk is significantly lower than that of non ACEI/ARB (5). Even studies have found that antihypertensive drugs targeting angiotensin converting enzyme can alleviate the inflammatory response and apoptosis of SARS-CoV-2 infected myocardial cells. Pharmacological RAS blockers, especially ARB, can regulate systemic and tissue RAS while increasing the expression and activity of ACE2 in experimental models (6).In the multicenter, blinded, and placebo-controlled randomized clinical trial, 205 hospitalized participants with COVID-19 and acute lung injury not already using RAAS inhibitors were randomized to oral losartan at the maximum dose approved by the US Food and Drug Administration to test the impact of drugs on disease outcomes. Compared with placebo, the 30 day mortality rate in the treatment group was significantly reduced (4.3% vs. 22.5%) (88). There is an urgent need for clinical evidence to determine the relative benefits and risks associated with the use of these drugs.

## 6 Potential treatment options

Receptors determine the invasion and transmission of viruses, as well as the clinical symptoms of patients. Therefore, functional receptors are key factors in preventing and treating viral diseases. These suggest therapeutic strategies through ACE2, including blocking the receptor binding domain (RBD) of the virus S protein to prevent the binding of human ACE2 and SARS-CoV-2. In addition to this receptor binding domain blocking strategy, other possible treatment options may include local use of ACE2 derived peptides, small molecule inhibitors, ACE2 antibodies, or single chain antibody fragments targeting ACE2 (94).

Although vaccine development provides the first line of defense against SARS-CoV-2 infection with antimicrobial drugs, more targeted interventions will provide alternative options. With the discovery that the receptor of SARS CoV-2 virus is ACE2, considering that the significant SARS CoV-2 related risk factors of hospitalization and death of patients with metabolic diseases (including obesity, hypertension, cardiovascular disease and diabetes) may reflect the overall activation of RAS system. It should be considered to regulate RAS activation through ACE2/Ang 1-7/MASR pathway to treat this disease. Clinical observational studies have also shown that in most cases, respiratory distress occurs many days after infection (usually about 14 days), indicating that this may not be a direct impact of initial viral infection, but rather a host response to ACE2 dysfunction and Ang II/ACE2 pathway imbalance, as well as host protease activation. Therefore, activating the downstream pathway of ACE2/Ang 1-7/MASR axis to induce ACE2 may prove to be a useful strategy for preventing lung and cardiovascular damage associated with SARS-CoV-2 infection (95).

Therefore, rebalancing RAS can mediate protection strategies. Two methods have emerged in this regard: using recombinant human soluble ACE2 as the “bait” receptor for the virus, or designing and applying ACE2 spike protein binding inhibitors based on structural biology of known virus ACE2 interactions. Especially, clinical trials of sACE2 have shown hope, and even the possibility of developing recombinant proteins in the form of aerosol delivery is currently underway. This method seems to have the potential to prevent a series of virus mutations in the future (35, 96). Jia et al. (97)found that ACE2 KO hypertensive mice exhibited pro-inflammatory cytokines and IL-1 β, IL6, TNF-α Enhancement of chemokines and administration of rhACE2 in humans can rescue Ang II induced T lymphocyte mediated inflammation,this also provides a theoretical basis for the treatment of diseases.

The plasma level of soluble ACE2 increases with age (98), especially elevated in critically ill COVID-19 patients (41). RhACE2 functionally isolates circulating viral particles to prevent interaction between S protein and endogenous ACE2, while regulating systemic RAS system balance. Promoting ACE2/Ang 1-7/MASR pathway conduction through rhACE2 or Ang 1-7 receptor agonist AVE0091 can have beneficial therapeutic effects on different causes of CVD and lung diseases. The Ang 1-7 receptor agonist AVE 0991 has been shown to have cardiorenal and lung protective effects, while rhACE2 has also been shown to improve acute lung injury, CVD, and kidney injury in various preclinical models. Maintaining ACE2 levels through negative regulation of ACE2/Ang 1-7/MASR can prevent the development of comorbidities in patients infected with SARS-CoV-2 (98).

Wysocki et al. (98) fused soluble ACE2 protein with albumin binding domain labels, which extended the duration of action of soluble ACE2 from approximately 8 h–96 h. The use of this engineered ACE2 protein as a substrate “bait” for COVID-19 virus may have beneficial effects in blocking virus entry into cells and limiting its replication, as well as restoring ACE2 activity and balancing angiotensin II and bradykinin. However, there is a potential issue with rhACE2, which is due to its large molecular size, which limits its activity and penetrance to tissue RAS, this will require us to study and solve in the future.

A recent study found that the Farneside X receptor (FXR) is a direct regulator of ACE2 transcription in several tissues affected by COVID-19, including the gastrointestinal and respiratory systems. Then we used the over-the-counter compound z-guggulsterone and the generic drug ursodeoxycholic acid (UDCA) to reduce FXR signaling and downregulate ACE2 in human lungs, bile duct cells, and intestinal organs, as well as corresponding tissues of mice and hamsters. Brevini et al. showed that UDCA mediated downregulation of ACE2 reduces susceptibility to SARS-CoV-2 infection *in vitro*, *in vivo*, and non *in situ* perfused human lungs and liver (99). This discovery suggests that some regulatory factors targeting ACE2 can affect the invasion of SARS virus.

## 7 New progress in ACE2 research

The new progress in ACE2 biology is the detection of ACE2 subtypes, with ACE107 being a truncated variant whose expression is upregulated in response to interferon therapy (14). This so-called δ subtype lacks functional catalytic sites and coronavirus spike protein binding sites (100). This mutation is particularly limited to the epithelium of the lung airways and the epithelium of the liver and bile ducts. Short ACE2 is substantially upregulated in response to interferon stimulation and rhinovirus infection, but not SARS-CoV-2 infection. This short isoform lacks SARS-CoV-2 spike high-affinity binding sites and, altogether, our data are consistent with a model where short ACE2 is unlikely to directly contribute to host susceptibility to SARS-CoV-2 infection. Whether the physiological role and expression of this subtype are directly related to COVID infection or disease prevention remains to be elucidated.

Another aspect of ACE2 biology that requires further research is the intracellular domain of ACE2 cleavage, which may serve as a transcription regulatory factor for intracellular or extracellular domain cleaved gap receptors similar to activation induced cell death (AICD), such as ADAM-17. This metabolic pathway may be a factor in the pathogenesis of COVID-19. A recent report indicates that after extracellular cleavage, ACE2 is released from the soluble C-terminal fragment (inner domain) of the protein γ Secretory enzyme like activity cleavage. Whether the intracellular fragment (ACE2-ICD) translocates to the nucleus that regulates transcription like AICD or is only degraded by proteasomes needs to be clarified (101). These findings add to the increasingly complex biology of ACE2. The possibility of epigenetic regulation of ACE2 transcription by AICD or related ICDs, similar to the regulation of vascular peptidase NEP, is also worth studying (75, 78).

## 8 Conclusion

ACE2 is an important channel for SARS-CoV-2 to invade the body, and it also participates in various complex pathological and physiological processes, including regulating the RAS system, apelin, KKS system, and immune system. What's more, because ACE2 is widely distributed in the human body, novel coronavirus infection can not only affect the respiratory system, but also directly invade the cardiovascular system through ACE2, causing or even aggravating cardiovascular diseases. In clinical practice, it is important for doctors to understand the possible complications of the cardiovascular system when treating COVID-19 patients. At the same time, the harm of viruses in different systems of the body presents opportunities and challenges for clinical medication. How can we control virus invasion while not exacerbating or even reducing basic cardiovascular diseases. Although vaccines targeting SARS-CoV-2 have been produced, the risk of disease remains high for individuals with weakened immunity. This requires us to continue exploring diseases and prepare ourselves for future pandemics.
