# Supplementary material for: The possible mechanism and research progress of ACE2 involved in cardiovascular injury caused by COVID-19: a review
Source: Front Cardiovasc Med. 2024 May 27;11:1409723. doi: 10.3389/fcvm.2024.1409723 (PMC11165996; doi:10.3389/fcvm.2024.1409723)
Supplement: Supplementary file 1 [file Datasheet1.pdf]

Supplementary Material

1 Supplementary Figures and Tables

1.1 Supplementary Figures

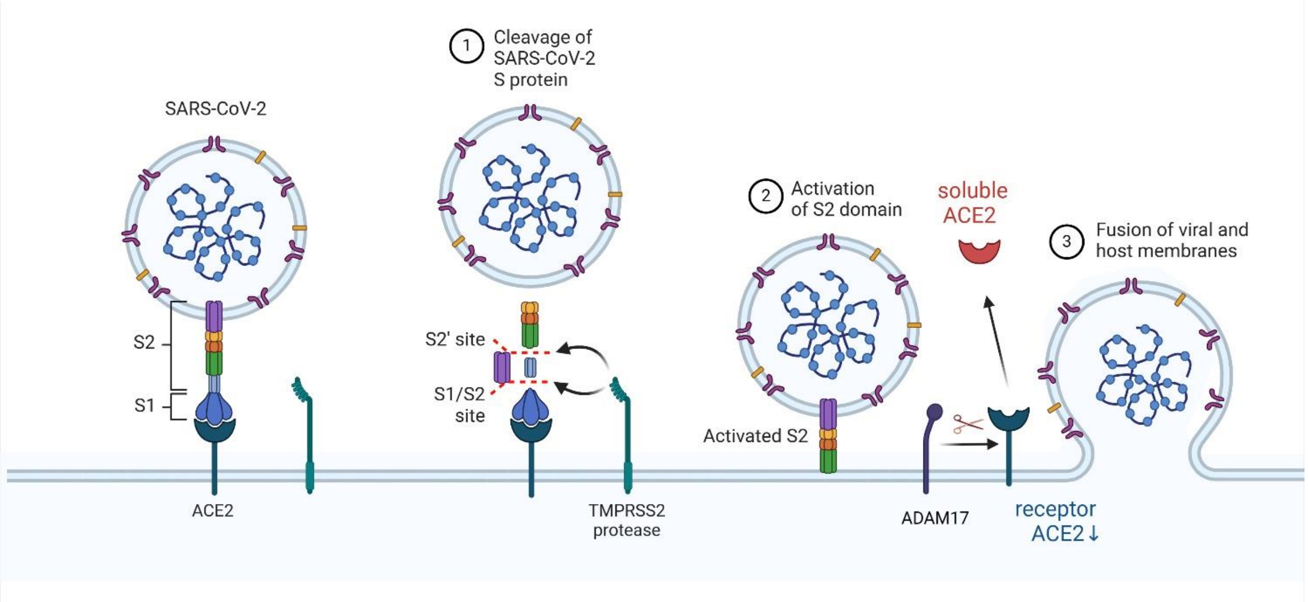

Supplementary Figure 1. ACE2 as a channel for SARS-CoV-2 virus to enter the body.

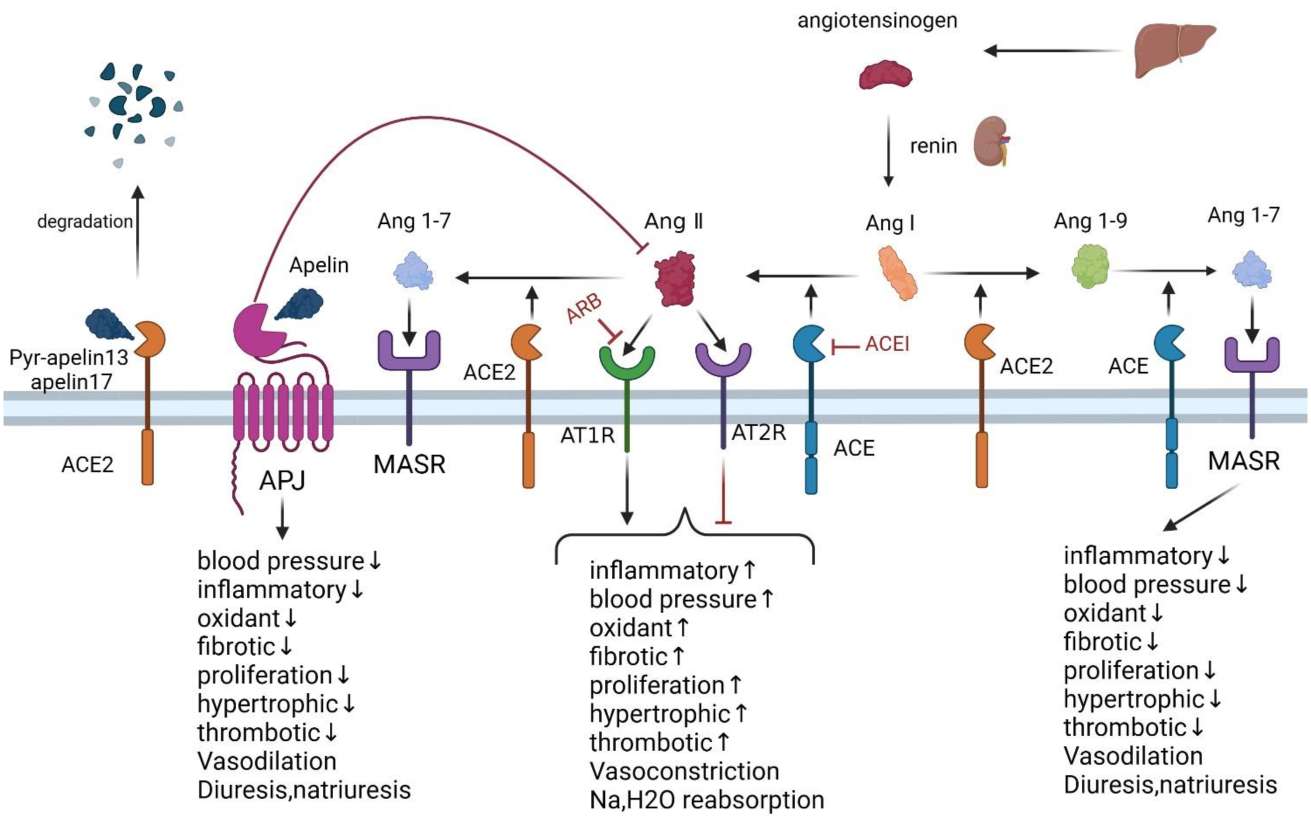

Supplementary Figure 2. ACE2 participates in regulating RAS system and apelin.

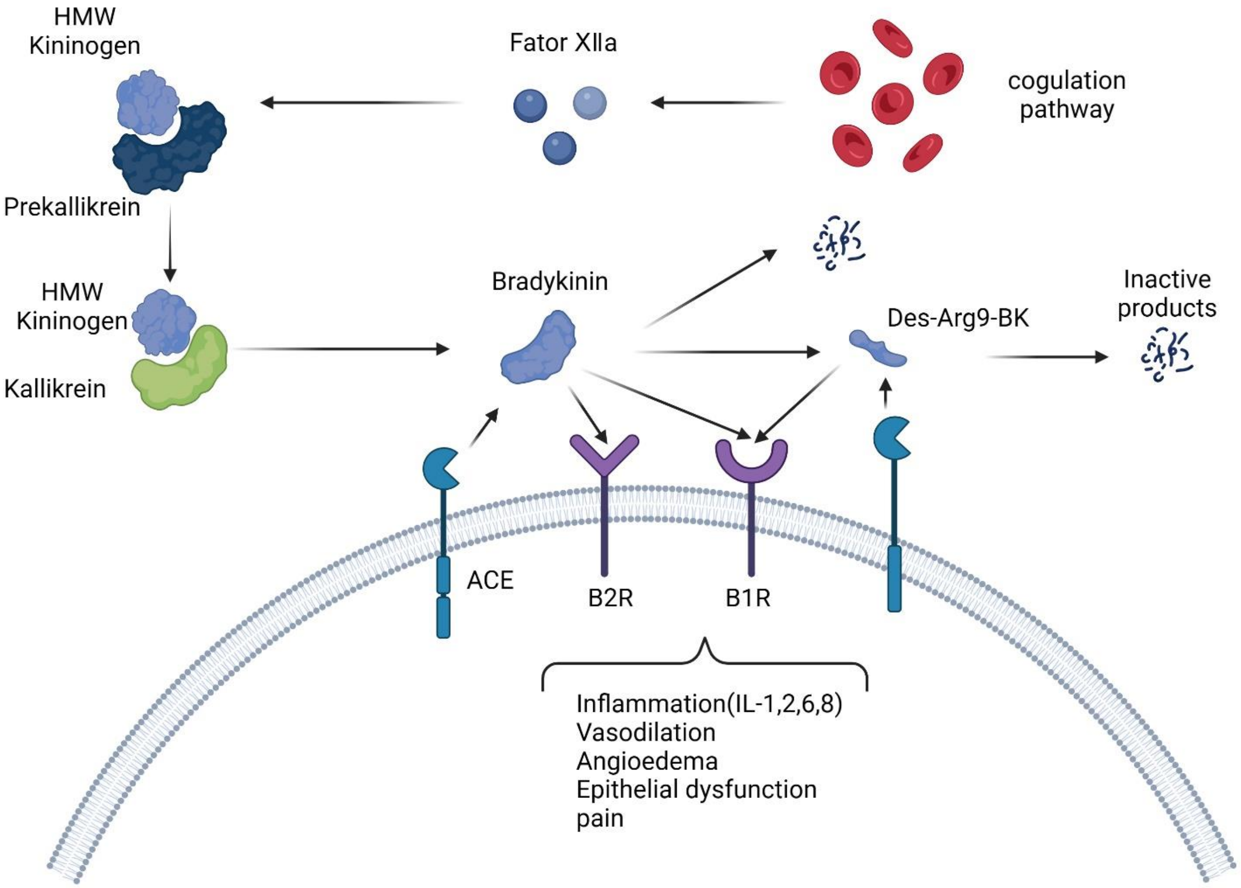

Supplementary Figure 3. ACE2 participates in KKS system.

Cardiovascular complications	Population	Morbidity (Total cases)	Reference
**Myocardial injury**	A healthy 53-year-old white woman	1	(50)
	A 43-year-old woman	1	(51)
	Patients from the Lazio region	60%(10)	(52)
	Patients from Zhongnan Hospital in Wuhan, China	7.2%(138)	(53)
	Patients from Tongji Hospital in Wuhan, China	14.67%(1159)	(54)
	Patients from Jinyintan Hospital and Wuhan Pulmonary Hospital	17%(191)	(55)
**Hypertension**	Cases of deaths at the Institute of Legal Medicine at the University Medical Center Hamburg-Eppendorf in Germany	43.6%(39)	(56)
	Patients from Jinyintan Hospital and Wuhan Pulmonary Hospital	30%(191)	(55)
	Chinese center for disease control and prevention	12.8%(44,672)	(57)
	Patients in the New York University (NYU)	43.6%(5894)	(4)
	Patients admitted in Italian hospitals.	54.9%(1591)	(58)
**Acute coronary syndrome**	Cases of deaths at the Institute of Legal Medicine at the University Medical Center Hamburg-Eppendorf in Germany	82%(39)	(56)
	Patients from Jinyintan Hospital and Wuhan Pulmonary Hospital	8%(191)	(55)
	Patients admitted in Italian hospitals.	13.6%(1591)	(58)
**Arrhythmias**	Patients at the Hospital of the University of Pennsylvania	7.6%(700)	(59)
	Patients from Zhongnan Hospital in Wuhan, China	16.7%(138)	(53)

	Patients with elevated levels of serum cardiac troponin I in Tongji Hospital in Wuhan, China	25.9%(170)	(54)
	Patients from the Lazio region	40%(10)	(52)
**Heart failure**	Patients from Jinyintan Hospital and Wuhan Pulmonary Hospital	23%(191)	(55)
	Cases of deaths at the Institute of Legal Medicine at the University Medical Center Hamburg-Eppendorf in Germany	28.2%(39)	(56)
	Patients admitted in Italian hospitals.	11.8%(1591)	(58)
**Coagulation defects**	Patients admitted to the Tongji Hospital in Wuhan,China	72%(183)	(60)
	Patients admitted to the ICU of 2 Dutch university hospitals and 1 Dutch teaching hospital.	31%(184)	(61)
	Patients from Jinyintan Hospital and Wuhan Pulmonary Hospital	19%(191)	(55)
	Patients from the Lazio region	60%(10)	(52)

Supplementary table 2. Clinical Study on SARS-CoV-2 Injury to the Cardiovascular System.
